# Receptor guanylyl cyclase B-mediated cGMP signalling controls axon bifurcation of cranial sensory ganglion neurons at the embryonic hindbrain

**DOI:** 10.1186/2050-6511-14-S1-P71

**Published:** 2013-08-29

**Authors:** Gohar Ter-Avetisyan, Fritz G Rathjen, Hannes Schmidt

**Affiliations:** 1 Developmental Neurobiology Group, Max Delbrück Center for Molecular Medicine, Berlin, Germany

## Background

**Figure 1:**
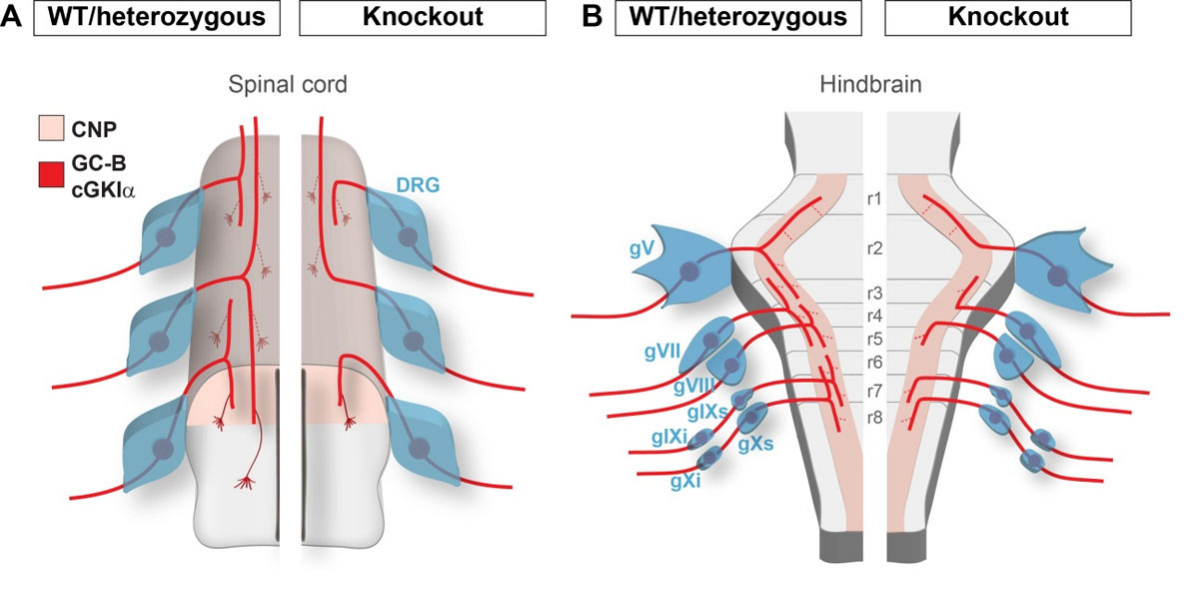
Scheme of branching errors observed in the absence of GC-B-mediated cGMP signalling in (A) DRG and (B) CSG neurons.

Axonal branching is a key principle for the establishment of neuronal circuitry that allows an individual neuron to innervate several target areas thus providing a physical framework for parallel processing of information. Our previous search for molecular determinants of axonal branching unravelled a cGMP signalling pathway in embryonic dorsal root ganglion (DRG) neurons composed of the ligand C-type natriuretic peptide (CNP), the receptor guanylyl cyclase B (GC-B, also termed Npr2), and the cGMP-dependent protein kinase Iα (cGKIα) that is essential for the bifurcation of sensory axons at the dorsal root entry zone of the spinal cord. Absence of any of these components causes a complete loss of bifurcation where sensory axons only turn in either a rostral or a caudal direction (Figure 1A). Consequently mutant mice reveal a reduced synaptic input onto second-order neurons in the dorsal horn of the spinal cord.

## Results

To examine whether the cGMP signalling system that underlies axon bifurcation in DRG neurons may also extend to other neuronal subpopulations we generated a GC-B-lacZ reporter mouse line for a detailed analysis of the GC-B expression pattern during embryonic development. A strong expression of GC-B that correlated with the localization of cGKIα was detected in neurons of all cranial sensory ganglia (CSG) at early embryonic stages when their central afferents enter the brainstem. Furthermore, a complimentary distribution of the ligand CNP at the entry zones of these axons in the hindbrain was observed.

To analyze the impact of GC-B-mediated cGMP signalling on axonal branching of CSG neurons we applied a genetic strategy for sparse labelling of GC-B-expressing neurons. For this purpose we generated a mouse mutant (GC-B-CreERT2) encoding a tamoxifen-inducible variant of Cre recombinase under control of the GC-B promoter that in combination with a conditional alkaline phosphatase reporter (Z/AP) enables the visualization of individual neurons within the genetically defined subset of GC-B-expressing cells. Comparing the branching patterns of GC-B heterozygous mice which resemble the wild-type phenotype with those of homozygous GC-B-deficient animals we found that in the absence of GC-B the bifurcation of axons of CSG neurons upon entering the hindbrain is impaired and only an ascending or a descending arm is formed (Figure 1B).

## Conclusion

Our data demonstrate that neurons of CSG - similar to DRG neurons - use GC-B-mediated cGMP signalling to trigger axonal bifurcation.

